# RAB42 is a Potential Biomarker that Correlates With Immune Infiltration in Hepatocellular Carcinoma

**DOI:** 10.3389/fmolb.2022.898567

**Published:** 2022-05-26

**Authors:** Hao Peng, Xuanlong Du, Yewei Zhang

**Affiliations:** ^1^ School of Medicine, Southeast University, Nanjing, China; ^2^ Hepatopancreatobiliary Center, The Second Affiliated Hospital of Nanjing Medical University, Nanjing, China

**Keywords:** HCC, immune infiltration, RAB42, CAFs, biomarker

## Abstract

**Backgrounds:** Hepatocellular carcinoma (HCC) is a malignant cancer with high mortality. Previous studies have reported that RAB42 is associated with prognosis and progression in glioma. However, the role of RAB42 in HCC is still unknown. Therefore, we aimed to elucidate the value of RAB42 in the predicting prognosis of HCC, and its relationship with immune cells infiltration.

**Methods:** UALCAN, HCCDB, and MethSurv databases were used to examine the expression and methylation levels of RAB42 in HCC and normal samples. cBioPortal and MethSurv were used to identify genetic alterations and DNA methylation of RAB42, and their effect on prognosis. The correlations between RAB42 and the immune cells and cancer-associated fibroblasts infiltration were analyzed by TIMER, TISIDB, and GEPIA database. The LinkedOmics database was used to analyze the enriched pathways associated with genes co-expressed with RAB42. EdU assay was used to evaluate the proliferation ability of liver cancer cells, and transwell assay was used to detect the invasion and migration ability of liver cancer cells.

**Results:** The expression levels of RAB42 were increased in HCC tissues than that in normal tissues. Highly expressed RAB42 was significantly correlated with several clinical parameters of HCC patients. Moreover, increased RAB42 expression clearly predicted poor prognosis in HCC. Furthermore, multivariate Cox regression analysis showed that RAB42 was an independent prognostic factor in HCC. The RAB42 genetic alteration rate was 5%. RAB42 DNA methylation in HCC tissues was lower than that in normal tissues. Among the 7 DNA methylation CpG sites, two were related to the prognosis of HCC. The results of gene set enrichment analysis (GSEA) showed that RAB42 was associated with various immune cells and cancer-associated fibroblasts infiltration in HCC. Meanwhile, we found RAB42 methylation was strongly correlated with immune infiltration levels, immunomodulators, and chemokines. Experiments *in vitro* indicated that knockdown of RAB42 inhibited the proliferation, invasion, and migration of liver cancer cells.

**Conclusions:** Our study highlights the clinical importance of RAB42 in HCC and explores the effect of RAB42 on immune infiltration in the tumor microenvironment, and RAB42 may act as a pro-oncogene that promotes HCC progression.

## Introduction

Hepatocellular carcinoma (HCC) is the most common type of primary liver cancer and the second most death-causing tumor after pancreatic cancer (Craig et al., 2020). Liver transplantation, hepatic resection, chemotherapy, and radiofrequency ablation can be used to intervene liver cancer. However, most HCC patients are diagnosed at an advanced stage, who do not benefit from these treatments (El-Serag et al., 2007). The tumor microenvironment (TME) is composed of various cells, including tumor stroma, endothelial cells, and infiltrating immune cells, including T cells, B cells, Dendritic cells (DCs), tumor-associated macrophages (TAMs), tumor-associated neutrophils (TANs), and myeloid-derived suppressor cells (MDSCs), natural killer cells (NK) et al. (Lu et al., 2019). Tumor-infiltrating immune cells interact with tumor cells to promote tumorigenesis, metastasis, and malignant progression (Lu et al., 2019). Currently, immunotherapy based on regulating the function of immune cells in the TME has arisen as an alternative option for the treatment of liver cancer (Liu H.-T. et al., 2021). The application of immune checkpoint inhibitors (ICI) can inhibit the function of suppressive immune cells by targeting the expression of checkpoint molecules, such as programmed death protein-1 (PD1), programmed death protein-ligand-1 (PD-L1), or cytotoxic T-lymphocyte-associated antigen 4 (CTLA4) on immune cells, thereby reactivating the anti-tumor immune response and killing tumor cells (He et al., 2020). Therefore, exploring the role of immune-related genes in the TME can provide a better understanding of the complexities of tumorigenesis and may provide new targets for immunotherapy of HCC.

RAB42 is a member of the Ras oncogene family that may involve in metabolism-related pathways (He et al., 2020). A recent study revealed that RAB42 expression in glioma was negatively related to the 5-year overall survival rate, which can be used as a risk factor to predict the poor prognosis of patients with glioma (Zhang et al., 2019). In addition, RAB42 correlated with multiple clinical characteristics and promoted the proliferation, migration, and invasion of glioma via activating VEGF signaling pathway (Liu B. et al., 2021). However, the role and mechanism of RAB42 on the prognosis, progression, DNA methylation, and immune infiltration in HCC have not been investigated.

**FIGURE 1:**
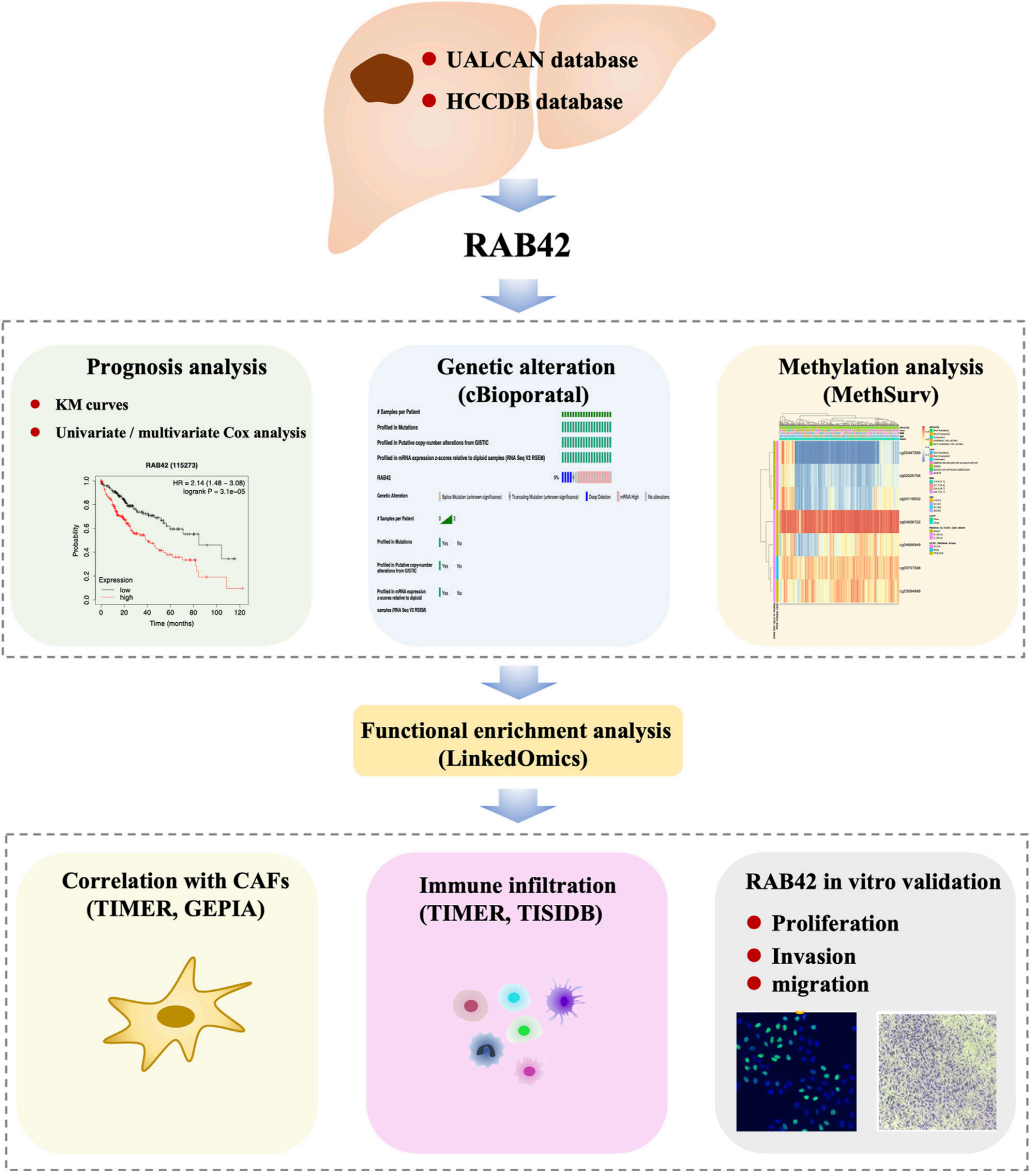
Workflow of this study.

In this study, by using multiple available databases, we aimed to explore the effects of RAB42 on HCC prognosis, immune infiltration, tumor proliferation, migration, and invasion. We found that RAB42 was significantly overexpressed in HCC and correlated with tumor stage, grade, and prognosis in HCC patients. Through the enrichment analysis of RAB42 co-expressed genes, it was shown that RAB42 may be involved in modulating the function of various immune cells and the methylation status of RAB42 may be associated with the immune infiltration in HCC. Finally, *in vitro* functional experiment was performed to validate the role of RAB42, and we found that RAB42 could promote the proliferation, invasion, and migration of HCC cells. Our study identified RAB42 may be used as a biomarker and a novel immune-related therapeutic target in HCC. The workflow of this study was illustrated in Figure 1.

## Materials and Methods

### UALCAN Analysis

To obtain the differential expression of RAB42 in HCC tissues and normal tissues, we used the UALCAN database for analysis (Chandrashekar et al., 2017). UALCAN is publicly available at http://ualcan.path.uab.edu. The methylation levels of RAB42 in the HCC were also analyzed by the UALCAN database. The differential expression of RAB42 and its promoter methylation status in patients with ages, tumor grade (grades 1, 2, 3, and 4), tumor stage (stages 1, 2, 3, and 4), and nodal metastasis were also compared.

### HCCDB Database

The HCCDB database (http://lifeome.net/database/hccdb/home.html) (Lian et al., 2018) contains 18 public datasets that include data on RAB42 mRNA expression in HCC. High RAB42 expression in HCC compared to adjacent liver tissues was validated using the HCCDB database.

### Prognostic Analysis in Public Databases

Kaplan-Meier plotter (http://kmplot.com/analysis/) was used to assess the relationship between clinic outcomes and RAB42 expression in HCC, such as Overall Survival (OS), Disease Specific Survival (DSS), Progress Free Survival (PFS), and Relapse Free Survival (RFS) and we calculated hazard ratios (HRs) of 95% confidence intervals and the log-rank *p*-value (Lanczky et al., 2021). Then the association between RAB42 expression and clinical characteristics was assessed using the ‘ggpubr’ package and Perl language. Moreover, we performed the univariate and multivariate Cox regression analyses to analyze the prognostic value of RAB42 in HCC.

### cBioPortal Analysis

The cBioPortal for cancer genomics (http://www.cbioportal.org) is a database of cancer genomics. Liver Hepatocellular Carcinoma (LIHC) (TCGA, Firehose Legacy) dataset including 379 samples was selected for subsequent analysis of the genomic profiles of RAB42. Meanwhile, Kaplan–Meier plots were created, and the log-rank test was performed to identify the significance of the difference between the survival curves, and differences with *p* < 0.05 were considered statically significant.

### MethSurv Database

MethSurv (https://biit.cs.ut.ee/methsurv/) is a web portal that provides survival analysis based on DNA methylation biomarkers using TCGA data. By using it, we analyzed the DNA methylation sites of RAB42 in the TCGA database. Moreover, the prognostic values of CpG methylation in RAB42 were also evaluated.

### LinkedOmics Database Analysis

LinkedOmics (http://www.linkedomics.org/login.php) is a publicly available portal that allows biologists and clinicians to access, analyze and compare cancer multi-omics data within and across tumor types (Vasaikar et al., 2018). By using the LinkFinder module, we identified the differentially co-expressed genes that correlated to RAB42 in HCC. The cancer cohort we selected was TCGA-LIHC, and the dataset was Firehose_RSEM_log2. The association results were analyzed by the Pearson correlation coefficient and visualized by volcano plot and heat maps. Kyoto Encyclopedia of Genes and Genomes (KEGG) pathway enrichment analysis and Gene Ontology (GO) analysis were used to investigate the potential function of RAB42.

### TIMER Database Analysis

TIMER, a comprehensive platform, is employed to analyze immune infiltration across multiple cancer types (https://cistrome.shinyapps.io/timer/). This web uses the TIMER algorithm to analyze the abundances of six tumor-infiltrating immune cells (B cells, CD4^+^ T cells, CD8^+^ T cells, neutrophils, macrophages, and dendritic cells) (Li T. et al., 2017). We used the TIMER website to evaluate the correlation of RAB42 with 6 types of tumor immune infiltrating cells. The levels of gene expression were calculated as log2 RSEM.

### TISIDB Database Analysis

TISIDB database (http://cis.Hku.hk/TISIDB/) is an integrated database for analyzing tumor cells and immune cells interplay (Ru et al., 2019). We analyze the correlation of RAB42 mRNA expression/methylation expression with tumor-infiltrating immune cells via this platform. The associations between RAB42 mRNA expression/methylation expression and immunomodulators, chemokines, and receptors were also analyzed by the TISIDB database.

### GEPIA2 Database Analysis

GEPIA2 (http://gepia2.cancer-pku.cn/), an updated and enhanced version of GEPIA, enables customers to compare the differentially expressed genes based on tumor and normal samples from the TCGA databases (Tang et al., 2019). By using the “Correlation Analysis” module in GEPIA2, we analyzed the correlation between RAB42 expression and some major markers of various immune cells.

### Cell Culture

Human liver cancer cell lines (SMMC7721, Huh7, MHCCLM3, MHCC97L, Hep3B, HepG2) and normal hepatic cells LO2 were purchased from the Shanghai Institute for Biological Science, Chinese Academy of Science (Shanghai, China). All cells were cultured in Dulbecco’s modified Eagle’s medium (DMEM, Gibco, Thermo Fisher Scientific, United States) containing 10% fetal bovine serum (FBS, Invitrogen, United States) and 1% penicillin-streptomycin at 37°C in humidified air with 5% CO_2_.

### RNA Extraction and qRT-PCR

Total RNA from cultured liver cancer cell lines was extracted with Trizol reagent (Invitrogen, United States) according to the manufacturer’s instructions. 1 μg RNA samples were reverse transcribed into cDNA using a SweScript RT I First Strand cDNA Synthesis Kit (Servicebio, China). The RAB42 primer sequences used for qRT-PCR were ‘RAB42 Forward: AGA​CCT​CGG​TTA​AAA​ACA​ACT​GC; RAB42 Reverse: AGC​CCT​CTT​CTA​GCT​TGA​TGT​C.’ GAPDH was used as a loading control, and the expression levels of RAB42 were calculated using the 2-ΔΔCt method.

### siRNA Transfection

The RAB42 siRNAs and synthetic sequence-scrambled siNC (RIBOBIO, China) were transiently transfected into liver cancer cells using Lipofectamine 2000 (Invitrogen, United States). Cells were collected for further experiments after 48 h.

### Ethynyl Deoxyuridine Incorporation Assay

After being transfected with siRAB42 for 48 h, SMMC7721 and Hep3B cells were seeded into 96-well plates at a density of 2 × 10^4^ cells per well. After an additional 24 h of incubation, SMMC7721 and Hep3B were to undergo EdU incorporation assays using an EdU kit (Beyotime, China) based on the manufacturer’s instructions. The samples were visualized with a Zeiss Axio Observer microscope, and images were captured in at least three random fields for further analysis.

### Transwell Migration and Invasion Assays

The transwell chamber (Millipore, United States) was used for migration and invasion assays. For migration assay, 2 × 10^4^ cells were seeded to the top chamber without serum, and 10% FBS in DMEM was added to the bottom chamber. To evaluate cell invasion, the chamber was pre-coated with Matrigel (1:8, invitrogen, United States). Then, 2 × 10^4^ cells were seeded in the upper chamber without serum, and the lower chamber was full of DMEM with 10% FBS. After 24 h, the cells that migrated or invaded through the membrane were fixed with 4% paraformaldehyde for 15 min and stained with crystal violet for 15 min at room temperature. The migration or invasion cells were photographed by a light microscope (Leica, Germany).

### Statistical Analysis

Statistical analysis was performed using the GraphPad Prism version 8.0 (GraphPad software, United States). Data are presented as mean ± SD. Two-tailed Student’s t test and one-way ANOVA were used to compare the difference for two or more groups. The Wilcoxon rank-sum test was used to compare differences between pairs of groups. Spearman correlation analysis was used to assess the significance of correlations. cBioPortal was used to examine the mutation and CNV profiles. Survival analysis (OS, PFS, DDS, RFS) were performed by Kaplan–Meier plots, and the differences were compared using the log-rank test. The Cox proportional hazards regression models were used to analyze the univariate and multivariable analyses. A two-tailed *p* value of 0.05 was considered statistically significant.

## Results

### Higher RAB42 Expression Levels in Hepatocellular Carcinoma Than in Normal Tissues

**FIGURE 2:**
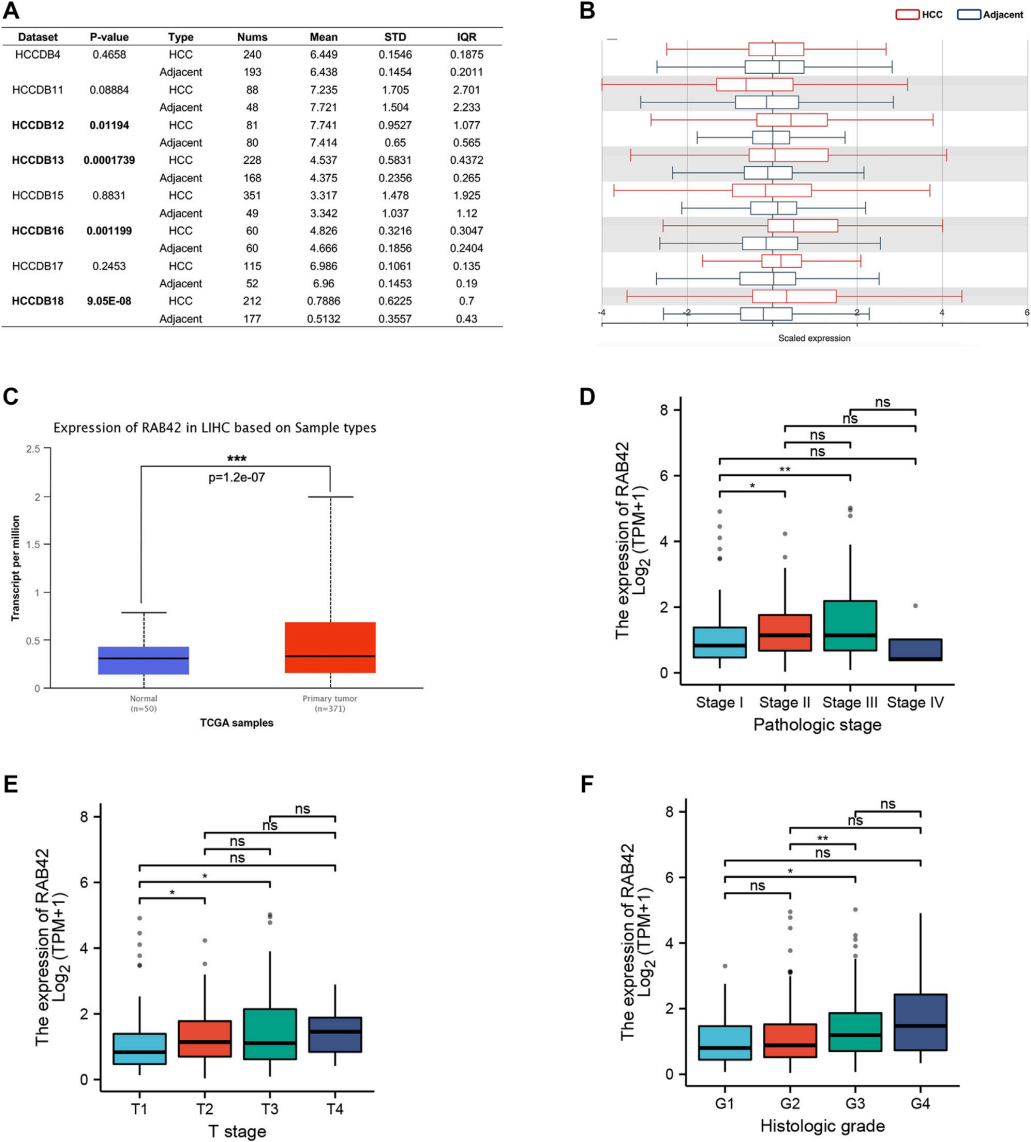
The expression level of RAB42 in HCC and its relationship with individual clinical parameters. **(A)** The table and **(B)** histogram listed eight HCC cohorts in the HCCDB database, and RAB42 was significantly highly expressed compared to the adjacent liver tissues in four of these datasets, namely HCCDB12 and HCCDB13, HCCDB16, HCCDB18. **(C)** Analysis using the UALCAN database also showed that RAB42 had higher expression in HCC compared to normal tissues. The expression of RAB42 significantly increased with advancing stage **(D)**, increasing T stage **(E)** and histologic grade. **(F)** *p < 0.05, **p < 0.01, ***p < 0.001, ns, no significance; HCC, hepatocellular carcinoma.

To explore the potential role of RAB42 in HCC, we first analyzed the expression data from the various database. Four HCC cohorts in the HCCDB database showed that the RAB42 mRNA level was significantly increased in HCC compared with adjacent liver tissue, namely HCCDB12 (*p* = 0.01194), HCCDB13 (*p* = 1.739E−04), HCCDB16 (*p* = 1.199E−03), and HCCDB18 (*p* = 9.05E−08) (Figures 2A,B). The results of UALCAN database analysis further confirmed that RAB42 mRNA expression was higher in HCC than that in normal tissues (Figure 2C). Additionally, we explored the relationship between RAB42 expression and individual clinical parameters in HCC. The 374 HCC patients with clinical information from the TCGA were divided into two groups with high and low RAB42 expression according to the median expression value of RAB42. Notably, RAB42 expression was associated with patients’ AFP levels (*p* = 0.0211), tumor stage (*p* = 6.00E−04), T stage (*p* = 0.0016) and histologic grade (*p* = 0.0015) in HCC, as illustrated in Table 1. Moreover, the expression of RAB42 also increased in higher stage and grade of HCC (Figures 2D–F). Collectively, these results suggested that RAB42 was highly expressed in HCC and may be related to the malignant progression of HCC.

**TABLE 1:** Correlations between the RAB42 expression and clinical characteristics of patients with HCC from the TCGA.

Characteristic	Total	High expression	Low expression	*p* value
Age				0.3547
≤65	235 (62.83%)	122 (65.24%)	113 (60.43%)	
>65	138 (36.9%)	64 (34.22%)	74 (39.57%)	
Gender				0.825
female	121 (32.35%)	62 (33.16%)	59 (31.55%)	
male	253 (67.65%)	125 (66.84%)	128 (68.45%)	
**AFP**				**0.0211**
high	117 (31.28%)	66 (35.29%)	51 (27.27%)	
normal	163 (43.58%)	68 (36.36%)	95 (50.8%)	
**Stage**				**6.00E−04**
stage I	173 (46.26%)	68 (36.36%)	105 (56.15%)	
stage II	87 (23.26%)	53 (28.34%)	34 (18.18%)	
stage III	85 (22.73%)	51 (27.27%)	34 (18.18%)	
stage IV	5 (1.34%)	1 (0.53%)	4 (2.14%)	
**T**				**0.0016**
T1	183 (48.93%)	73 (39.04%)	110 (58.82%)	
T2	95 (25.4%)	58 (31.02%)	37 (19.79%)	
T3	80 (21.39%)	47 (25.13%)	33 (17.65%)	
T4	13 (3.48%)	8 (4.28%)	5 (2.67%)	
M				0.6249
M0	268 (71.66%)	134 (71.66%)	134 (71.66%)	
M1	4 (1.07%)	1 (0.53%)	3 (1.6%)	
N				0.5496
N0	254 (67.91%)	120 (64.17%)	134 (71.66%)	
N1	4 (1.07%)	3 (1.6%)	1 (0.53%)	
**Grade**				**0.0015**
G1	55 (14.71%)	21 (11.23%)	34 (18.18%)	
G2	178 (47.59%)	78 (41.71%)	100 (53.48%)	
G3	124 (33.16%)	79 (42.25%)	45 (24.06%)	
G4	12 (3.21%)	7 (3.74%)	5 (2.67%)	

Bold means the p-value for that parameter is statistically significant.

### The Prognostic Value of RAB42

**FIGURE 3:**
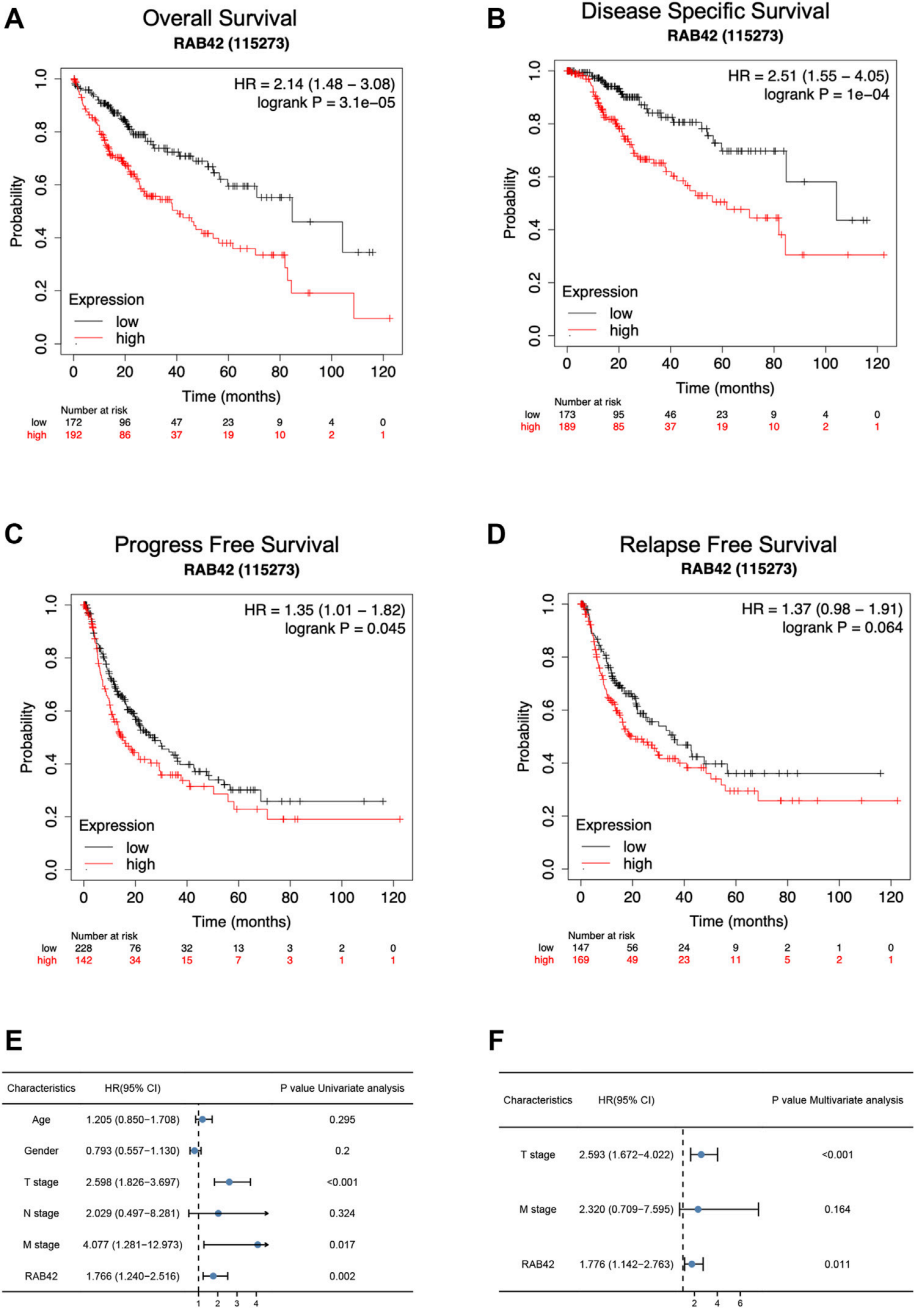
The survival analysis and the prognostic value of RAB42 in HCC. **(A** **–** **D)** The OS **(A)**, DSS **(B)**, PFS **(C)**, and RFS **(D)** survival curves comparing patients with high (red) and low (black) RAB42 expression in HCC were plotted using the Kaplan–Meier plotter database at the threshold of the *p*-value of <0.05. **(E)** Univariate Cox regression analyses of OS-related factors in HCC from TCGA dataset. **(F)** Multivariate Cox regression analyses of OS-related factors in HCC from TCGA dataset. HCC, hepatocellular carcinoma; OS, overall survival; DSS, disease specific survival; PFS, progress free survival; RFS, relapse free survival.

According to the Kaplan–Meier survival curves, HCC cases with higher RAB42 expression showed poor overall survival (OS) [hazard ratio (HR) = 2.14, *p* = 3.1e−05], disease specific survival (DSS) [hazard ratio (HR) = 2.51, p = 1e−04] and a worse progress free survival (PFS) [hazard ratio (HR) = 1.35, *p* = 0.045] (Figures 3A–C). In addition, we also analyzed the relapse free survival (RFS) in HCC patients, the median survival of high RAB42 expression was 21.23 months, and the low RAB42 expression was 36.1 months, however, it may be the insufficient number of tracked patients leading to no significant difference in statistical results [hazard ratio (HR) = 1.37, *p* = 0.064] (Figure 3D). Univariate Cox regression analyses showed a correlation between OS with T stage [hazard ratio (HR) = 2.598, *p* < 0.001], M stage [hazard ratio (HR) = 4.077, *p* = 0.017] and RAB42 expression [hazard ratio (HR) = 1.766, *p* = 0.002] (Figure 3E). Furthermore, multivariate Cox regression analysis revealed that RAB42 expression (HR = 1.776, *p* = 0.011), T stage (HR = 2.593, *p* < 0.001) were independent prognostic indicators for HCC patients (Figure 3F). Overall, these findings indicated that RAB42 is a hazard risk factor for predicting worse prognosis in HCC.

### RAB42 Genetic Alteration in Patients With Hepatocellular Carcinoma

**FIGURE 4:**
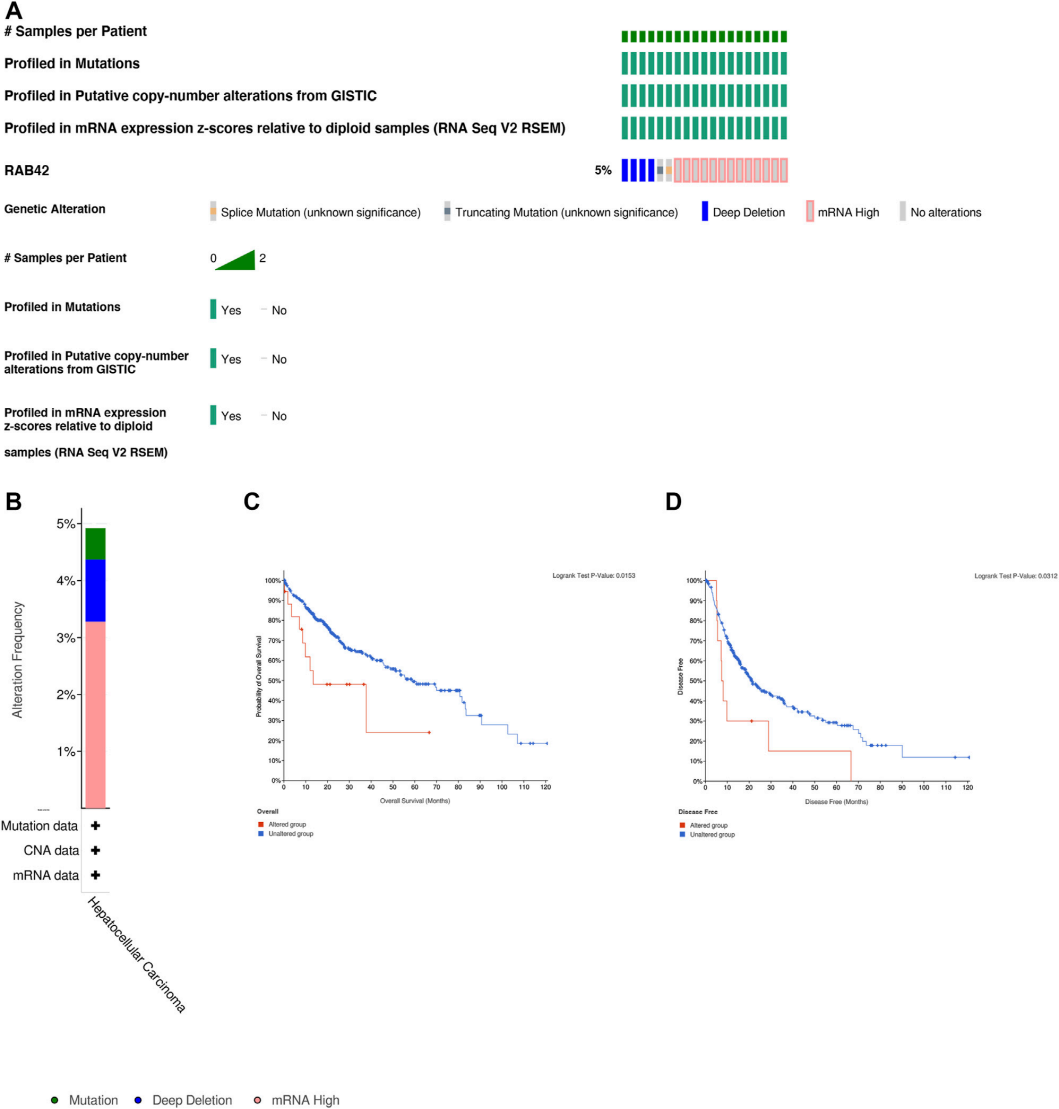
Genetic alteration of RAB42 in HCC. **(A)** OncoPrint summarized the genetic alteration in RAB42 based on LIHC (TCGA, Firehose Legacy, 379 samples) from cBioPortal database. **(B)** The histogram showed the gene alteration frequency of RAB42 from TCGA. **(C)** Kaplan-Meier analysis showed genetic alterations in RAB42 were related to shorter overall survival (*p* = 0.0153) in HCC patients. **(D)** Kaplan-Meier analysis showed genetic alterations in RAB42 were related to shorter disease free survival (*p* = 0.0312) in HCC patients.

Analysis of genetic alteration data based on LIHC (TCGA, Firehose Legacy, 379 samples) from cBioPortal database revealed the percentage of RAB42 genetic alterations in HCC was 5% (Figure 4A). The histogram showed a summary of the different types of genetic alterations of RAB42 in HCC samples (Figure 4B). Additionally, we explored the association between genetic alteration of RAB42 and clinical survival in HCC. The Kaplan–Meier plots and log-rank tests indicated that the HCC cases with altered RAB42 showed poor OS (*p* = 0.0153) and DFS (*p* = 0.0312) compared with the unaltered group (Figures 4C,D). These results suggested that genetic alteration of RAB42 in HCC was involved in mRNA expression alteration and poor clinical survival, which deserved further mechanism research.

### RAB42 Methylation and Prognostic Value in Hepatocellular Carcinoma

**FIGURE 5:**
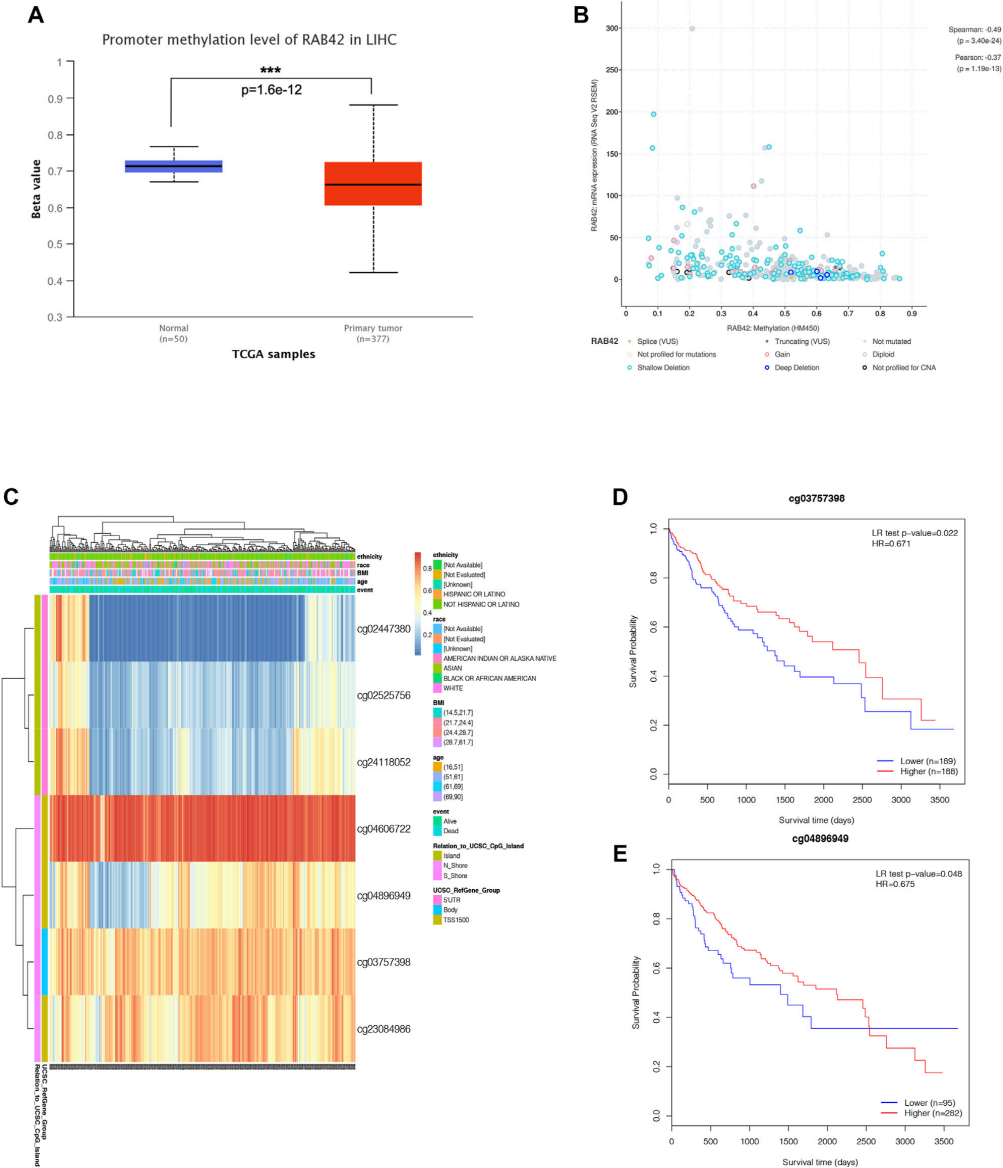
DNA methylation expression level of RAB42 in HCC. **(A)** Promoter methylation level of RAB42 in HCC and normal tissues were analyzed with TCGA data by using UALCAN database. **(B)** Correlation between RAB42 mRNA expression and DNA methylation level was analyzed with TCGA data by the cBioPortal database (Spearman’s correlation r = −0.49, *p*-value = 3.40E−24). **(C)** The heat map showed the RAB42 DNA methylation at CpG sites by using the MethSurv database. **(D)** HCC patients with lower RAB42 methylation of cg03757398 CpG sites had a worse overall survival than those with higher RAB42 methylation (HR = 0.671, *p* = 0.022). **(E)** HCC patients with lower RAB42 methylation of cg04896949 CpG sites had a worse overall survival than those with higher RAB42 methylation (HR = 0.675, *p* = 0.048).

Accumulative evidence has shown that DNA methylation, as a major epigenetic modification mechanism, plays an essential role in the development and progression of HCC (Anwar et al., 2014). Therefore, we wanted to explore whether the methylation level of RAB42 affected its expression and prognosis of HCC. Using the UALCAN database, we found that the methylation level of the RAB42 promoter region was significantly lower in HCC than in normal tissues (Figure 5A). Subsequently, we explored the correlation between RAB42 promoter methylation and individual clinical parameters. The results revealed that RAB42 methylation status was significantly related to patients’ age (*p* = 8.0E−04) and AFP levels (*p* = 0.019) (Table 2). Scatter plots also showed that RAB42 methylation was negatively correlated with RAB42 mRNA expression (Spearman, r = −0.49, *p* = 3.40e−24) (Figure 5B). Among 7 predicted CpG sites of RAB42, cg03757398 and cg04896949 were significantly correlated with the prognosis of HCC (Figure 5C) (Table 3). Patients with low RAB42 methylation of these CpG sites had a worse overall survival than patients with high RAB42 methylation (Figures 5D,E). These results revealed that the methylation levels of RAB42 act as an effective prognostic biomarker for HCC, indicating RAB42 may play a critical role in HCC progression.

**TABLE 2:** The association between RAB42 promoter methylation level and clinical characteristics of patients with HCC from the TCGA.

Characteristic	Total	High Methylation	Low Methylation	*p* value
**Age**				**8.00E**−**04**
≤65	235 (62.83%)	101 (54.01%)	134 (71.66%)	
>65	138 (36.9%)	85 (45.45%)	53 (28.34%)	
Gender				0.5072
female	121 (32.35%)	57 (30.48%)	64 (34.22%)	
male	253 (67.65%)	130 (69.52%)	123 (65.78%)	
**AFP**				**0.019**
high	117 (31.28%)	50 (26.74%)	67 (35.83%)	
normal	163 (43.58%)	94 (50.27%)	69 (36.9%)	
Stage				0.3147
stage I	173 (46.26%)	94 (50.27%)	79 (42.25%)	
stage II	87 (23.26%)	45 (24.06%)	42 (22.46%)	
stage III	85 (22.73%)	36 (19.25%)	49 (26.2%)	
stage IV	5 (1.34%)	2 (1.07%)	3 (1.6%)	
T				0.3743
T1	183 (48.93%)	99 (52.94%)	84 (44.92%)	
T2	95 (25.4%)	47 (25.13%)	48 (25.67%)	
T3	80 (21.39%)	34 (18.18%)	46 (24.6%)	
T4	13 (3.48%)	6 (3.21%)	7 (3.74%)	
M				0.7214
M0	268 (71.66%)	125 (66.84%)	143 (76.47%)	
M1	4 (1.07%)	1 (0.53%)	3 (1.6%)	
N				1
N0	254 (67.91%)	119 (63.64%)	135 (72.19%)	
N1	4 (1.07%)	2 (1.07%)	2 (1.07%)	
Grade				0.3677
G1	55 (14.71%)	27 (14.44%)	28 (14.97%)	
G2	178 (47.59%)	96 (51.34%)	82 (43.85%)	
G3	124 (33.16%)	54 (28.88%)	70 (37.43%)	
G4	12 (3.21%)	6 (3.21%)	6 (3.21%)	

Bold means the p-value for that parameter is statistically significant.

**TABLE 3:** The significant prognostic values of CpG in RAB42.

Gene symbol	CpG name	Hazard Ratio	CI	LR test *p* value	UCSC Ref gene group	Relation to UCSC CpG Island
**RAB42**	cg02447380	1.14	(0.784; 1.659)	0.5	5′UTR	Island
	cg02525756	0.86	(0.583; 1.27)	0.45	5′UTR	Island
	**cg03757398**	**0.671**	**(0.476**; **0.945)**	**0.022**	**Body**	**S_Shore**
	cg04606722	0.838	(0.567; 1.239)	0.38	TSS1500	N_Shore
	**cg04896949**	**0.675**	**(0.463**; **0.985)**	**0.048**	**TSS1500**	**N_Shore**
	cg23084986	1.167	(0.829; 1.643)	0.38	TSS1500	N_Shore
	cg24118052	0.7	(0.483; 1.016)	0.067	5′UTR	Island

Bold means the p-value for that parameter is statistically significant.

### Functional Enrichment Analysis of RAB42 Co-expressed Genes in Hepatocellular Carcinoma

**FIGURE 6:**
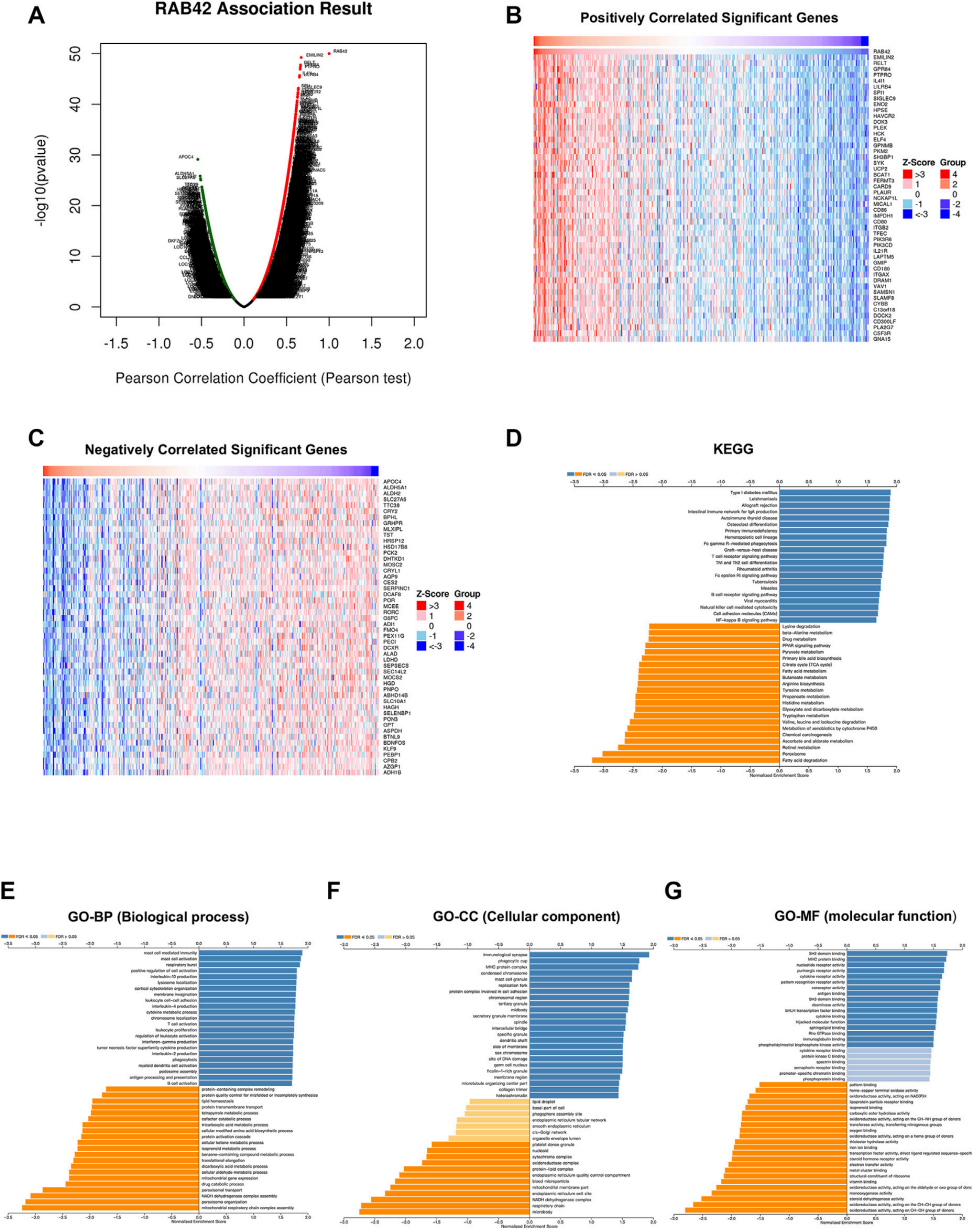
RAB42 co-expressed genes and KEGG, GO enrichment analysis in HCC patients. **(A)** Volcano plots showed the differentially co-expressed genes of RAB42 in HCC by the LinkedOmics database. **(B**,**C)** The heatmap showed the top 50 positively **(B)** and negatively **(C)** differentially correlated genes of RAB42 in HCC by the LinkedOmics database. **(D)** KEGG enrichment analysis revealed diseases and signaling pathways related to RAB42 co-related genes. **(E** **–** **G)** RAB42 co-expressed genes were annotated by using GO analysis. KEGG, Kyoto Encyclopedia of Genes and Genomes; GO, Gene Ontology; BP, Biological Process; CC, Cellular Component; MF, Molecular Function.

To explore the potential mechanism by which RAB42 function in HCC, we identified 13,094 differentially co-expressed genes from the LinkedOmics database, including 9955 positively correlated genes and 3139 negatively correlated genes (Figure 6A). Heatmap showed the top 50 differentially co-expressed genes, which positively and negatively correlated with RAB42 (Figures 6B,C). Subsequently, we performed KEGG and GO enrichment analysis on these differentially co-expressed genes to explore the potential pathways of RAB42 in regulating liver cancer. The KEGG enrichment analysis illustrated that RAB42 co-expressed genes may be involved in various immune-related diseases and pathways, such as allograft rejection, autoimmune thyroid disease, and graft-versus-host disease, T cell/B cell receptor signaling pathway, Th1 and Th2 cell differentiation, and natural killer cells mediated cytotoxicity (Figure 6D). According to the GO Biological process analysis, activation of different immune cells and production of immune-related cytokines were the most enriched categories, such as mast cell activation, T/B cell activation, and myeloid dendritic cell activation, in terms of cytokines, related to the enrichment of IL-10, IL-4, IL-2, and tumor necrosis factor superfamily cytokine production (Figure 6E). At the cellular component level, immunological synapse, phagocytic cup, and MHC protein complex were the most enriched categories (Figure 6F). At the molecular level, SH2 domain binding, MHC protein binding, and nucleotide/purinergic/cytokines/pattern recognition receptors showed obvious enrichment (Figure 6G). All these results revealed that RAB42 expression may influence the immune microenvironment in HCC.

### Correlation Between RAB42 Expression and Immune Cell Infiltration in Hepatocellular Carcinoma

**FIGURE 7:**
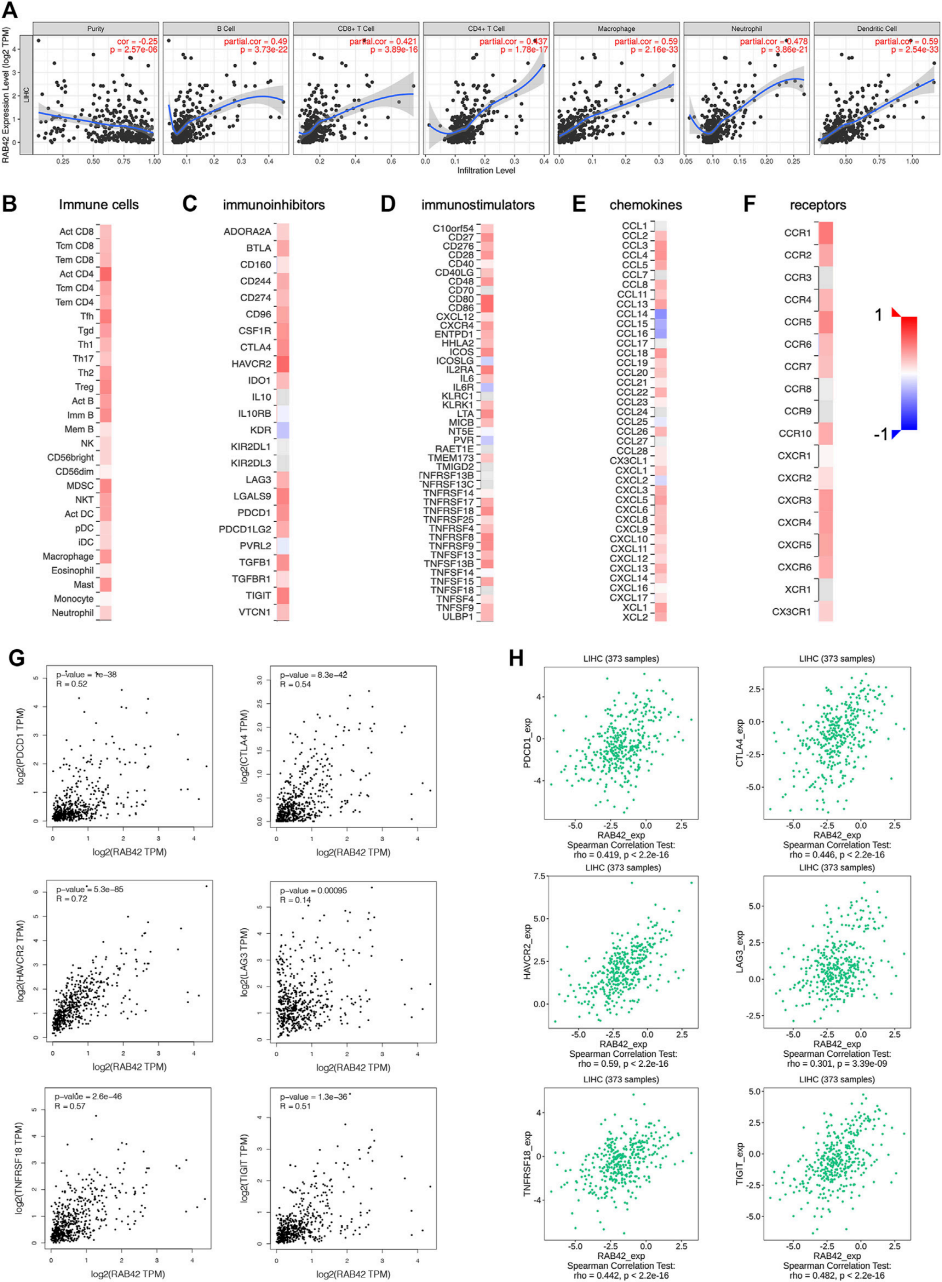
Relationship between RAB42 expression and immune cells infiltration in HCC. **(A)** Correlation analysis based on TIMER database showed RAB42 expression was positively correlated with tumor infiltrating lymphocytes in HCC tissues, including B cells, CD8^+^ T cells, CD4^+^ T cells, macrophages, neutrophils, and dendritic cells. Heat map showed the correlation between the RAB42 expression and various types of immune cells **(B)**, immunoinhibitors **(C)**, immunostimulators **(D)**, chemokines **(E)**, receptors **(F)** in HCC from the TISIDB database. The GEPIA database **(G)** and the TISIDB database **(H)** were used to evaluate the relationship between RAB42 expression and major immune checkpoint molecules in HCC, including PDCD1, CTLA-4, HAVCR2, LAG3, TNFRSF18 and TIGIT.

Based on the results of the above enrichment analysis, we wanted to explore the relationship between RAB42 expression and immune infiltration in HCC. After adjustment based on tumor purity using TIMER database, RAB42 expression was positively correlated with infiltrating levels of B cells (r = 0.49, *p* = 3.73e−22), CD8^+^ T cells (r = 0.421, *p* = 3.89e−16), CD4^+^ T cells (r = 0.437, *p* = 1.78e−17), macrophages (r = 0.59, *p* = 2.16e−33), neutrophils (r = 0.478, *p* = 3.86e−21), and DCs (r = 0.59, *p* = 2.54e−33) in HCC but negatively correlated with tumor purity (r = −0.25, *p* = 2.57e−06) (Figure 7A). Furthermore, we employed the GEPIA database to verify the relationship between the RAB42 expression and various immune cells markers in HCC. As listed in Table 4, RAB42 significantly positively correlated with the gene markers of CD8^+^ T cells, Th1 cells, Th2 cells, Tfh cells, Th17 cells, Treg cells, tumor-associated macrophages (TAM), M1 macrophages, M2 macrophages, neutrophils cells, NK cells, DCs and B cells. Using the TISIDB database, we also confirmed that RAB42 was positively correlated with immune cells infiltration (Figure 7B). The correlation between RAB42 and various types of immune cells was evaluated through the TISIDB database (Table 5). Additionally, RAB42 was significantly correlated with immunoinhibitors (Figure 7C), immunostimulators (Figure 7D), chemokines (Figure 7E), and receptors (Figure 7F). We noticed that RAB42 was significantly associated with some anti-tumor immune cells, such as CD8^+^ T cells. Considering the immunosuppressive microenvironment of HCC, we speculated that RAB42 could induce effector T cells exhaustion. The relationship of RAB42 with immune checkpoint molecules such as programmed cell death 1 (PDCD1), cytotoxic T-lymphocyte antigen 4 (CTLA-4), hepatitis A virus cellular receptor 2 (HAVCR2), lymphocyte activating gene 3 protein (LAG3), TNF receptor superfamily member 18 (TNFRSF18) and T cell immunoreceptor with Ig and ITIM domains (TIGIT) was assessed in both the GEPIA and TISIDB databases. The results showed that RAB42 was positively associated with these immune checkpoint molecules (Figures 7G,H). In addition, RAB42 was also significantly associated with the expression of immunosuppressive cytokines such as IL10, TGF-β (Supplementary Figure S1A), which the presence of these cytokines could stimulate T cell dysfunction. Meanwhile, using the TIMER2 database, we found that RAB42 was significantly associated with Treg cells infiltration in HCC (Supplementary Figure S1B), which also created a favorable microenvironment for promoting T cell exhaustion. According to a recent study that divides T cell exhaustion status into four stages (Beltra et al., 2020), we analyzed the correlation of RAB42 with major genes in each stage, and the results showed that RAB42 was positively correlated with exhausted T cells in all stages (Supplementary Figure S1C). Collectively, RAB42 may suppress the immune response by recruiting immunosuppressive cells and inducing exhaustion of effector T cells, thereby forming an immunosuppressive microenvironment (TIME) to promote HCC progression.

**TABLE 4:** Correlation analysis between RAB42 expression and immune cell markers in HCC.

	Biomarker	Cor	*p* value
CD8+T	CD8A	0.44	***
	CD8B	0.49	***
Th1	T-bet (TBX21)	0.25	***
	STAT4	0.099	*
	STAT1	0.51	***
	IFN-g(IFNG)	0.23	***
	TNF-a(TNF)	0.4	***
Th2	GATA3	0.39	***
	STAT5A	0.51	***
	CCR3	0.28	***
Tfh	BCL6	0.0035	0.94
	IL21	0.2	***
	CXCR5	0.27	***
	ICOS	0.35	*
Th17	STAT3	0.074	0.091
	IL17A	0.037	0.39
	IL-21R	0.37	*
Treg	FOXP3	0.022	0.62
	CCR8	0.37	*
	TGFb(TGFB1)	0.34	***
	IL2RA	0.5	*
M1	INOS(NOS2)	0.022	0.61
	IRF5	0.3	***
	COX2(PTGS2)	0.015	0.72
M2	ARG1	−0.13	**
	CD206 (MRC1)	0.16	***
	CD115 (CSF1R)	0.63	***
N	CD66b(CEACAM8)	−0.073	0.093
	CD11b(ITGAM)	0.49	***
	CCR7	0.3	***
	FUT4	0.46	***
NK	CD7	0.38	***
	XCL1	0.34	***
	KIR3DL1	0.034	0.43
DC	HLA-DPB1	0.58	***
	HLA-DQB1	0.44	***
	HLA-DRA	0.58	***
	HLA-DPA1	0.58	***
	BDCA-1(CD1C)	0.38	***
	BDCA-4(NRP1)	0.46	***
	CD11c	0.48	***
B	CD19	0.33	***
	CD79A	0.43	***
TAM	CCL2	0.33	***
	CD68	0.63	***
	IL10	0.45	***

**TABLE 5:** The correlation between RAB42 expression and tumor lymphocyte infiltration in HCC (TISIDB).

	**LIHC**	
	**r**	**p**
Activated CD8 T cell	0.253	7.83E−07
Central memory CD8 T cell	0.27	1.36E−07
Effector memory CD8 T cell	0.284	2.64E−08
Activated CD4 T cell	0.576	2.20E−16
Central memory CD4T cell	0.356	1.77E−12
Effector memory CD4 T cell	0.313	7.60E−10
T follicular helper cell	0.501	2.20E−16
Gamma delta T cell	0.367	3.44E−13
Type 1 T helper cell	0.277	6.08E−08
Type 17 T helper cell	0.228	8.97E−06
Type 2 T helper cell	0.402	2.20E−16
Regulatory T cell	0.467	2.20E−16
Activated B cell	0.374	1.09E−13
Immature B cell	0.442	2.20E−16
Memory B cell	0.115	0.0269
natural killer cell	0.158	0.00226
CD56bright natural killer cell	0.164	0.00149
CD56dim natural killer cell	0.051	0.322
Myeloid derived suppressor cell	0.459	2.20E−16
Natural killer T cell	0.353	2.82E−12
Activated dendtritic cell	0.36	9.48E−13
Plasmacytoid dendtritic cell	0.157	0.0024
Immature dendtritic cell	0.167	0.00124
Macrophage	0.4	7.83E−17
Eosinophil	0.089	0.0852
Mast	0.417	2.20E−16
Monocyte	0.033	0.53
Neutrophil	0.182	0.000407

### Correlation Between RAB42 Expression and Cancer-Associated Fibroblasts Infiltration in Hepatocellular Carcinoma

**FIGURE 8:**
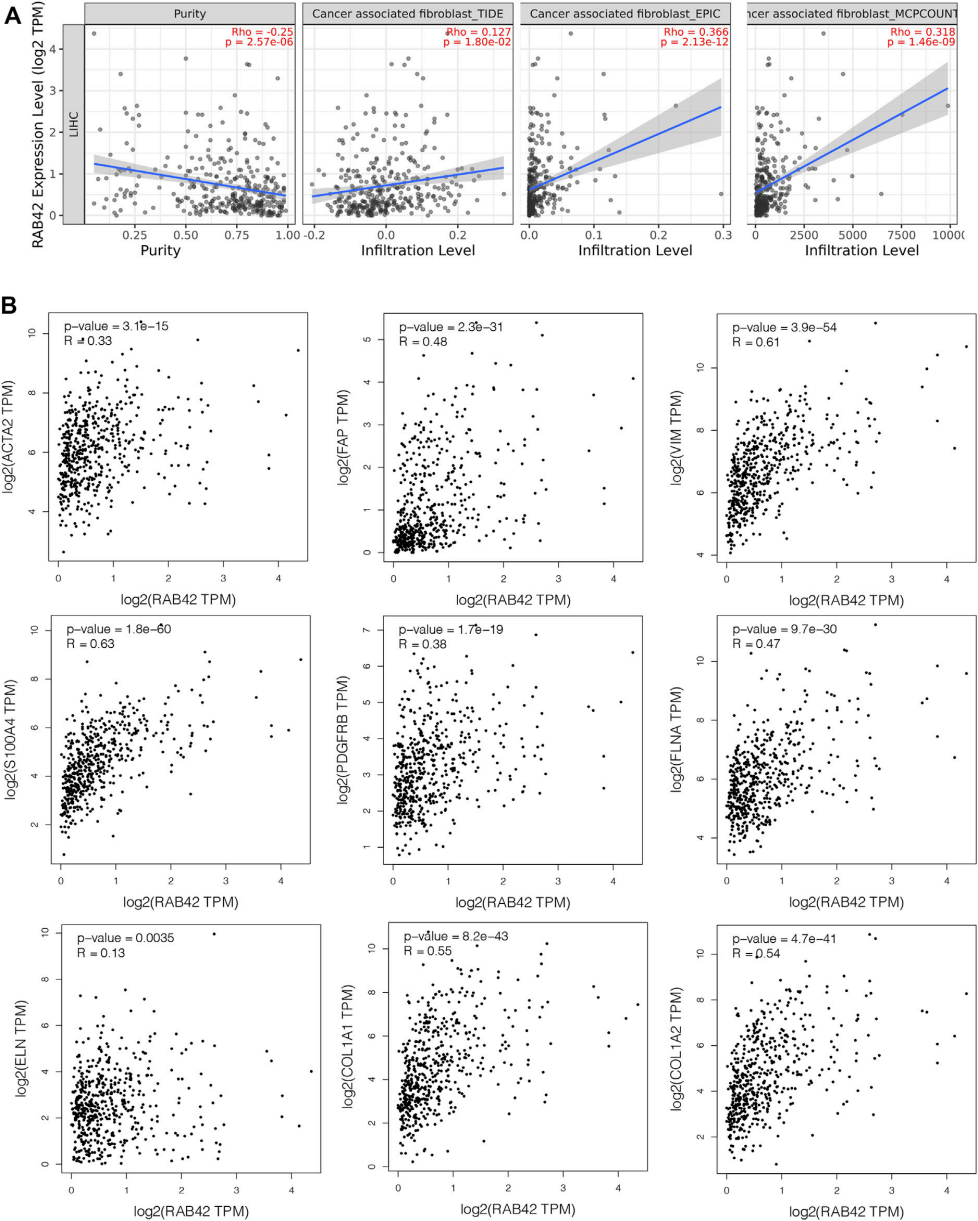
Correlation between RAB42 expression and CAFs in HCC. **(A)** The association between RAB42 expression and the infiltration level of CAFs was investigated by using various algorithms in HCC from the TCGA. **(B)** RAB42 expression was positively correlated with several markers of CAFs (ACTA2, FAP, VIM, S100A4, PDGFRB) and ECM-related genes (ELN, FLNA, COL1A1, COL1A2) based on GEPIA database. CAFs, cancer-associated fibroblasts; ECM, extracellular matrix.

Cancer-associated fibroblasts (CAFs) constitute the majority components of stroma in tumor and play multiple roles in facilitating tumor cells proliferation, migration, stemness and supporting the formation of TIME in HCC (Jia et al., 2021). We, therefore, investigated the possible association between the infiltration level of CAFs and the RAB42 expression in HCC. Using the TIDE, EPIC, and MCPCOUNTER algorithms, we found a statistically positive association between CAFs infiltration and RAB42 expression (Figure 8A). Subsequently, we used the GEPIA database to analyze the correlation of RAB42 with major CAFs markers (ACTA2, FAP, S100A4, VIM, PDGFRB) and extracellular matrix (ECM)-related genes (ELN, FLNA, COL1A1, COL1A2), and the results showed that RAB42 was positively correlated with these genes (Figure 8B). Collectively, these results suggested that the expression of RAB42 in HCC may promote the infiltration of CAFs, and CAFs may secret the abundant ECM proteins to form a high density of stroma in tumor tissue to promote HCC progression.

### Correlation Between RAB42 Methylation and Immunosuppressive Status in Hepatocellular Carcinoma

**FIGURE 9:**
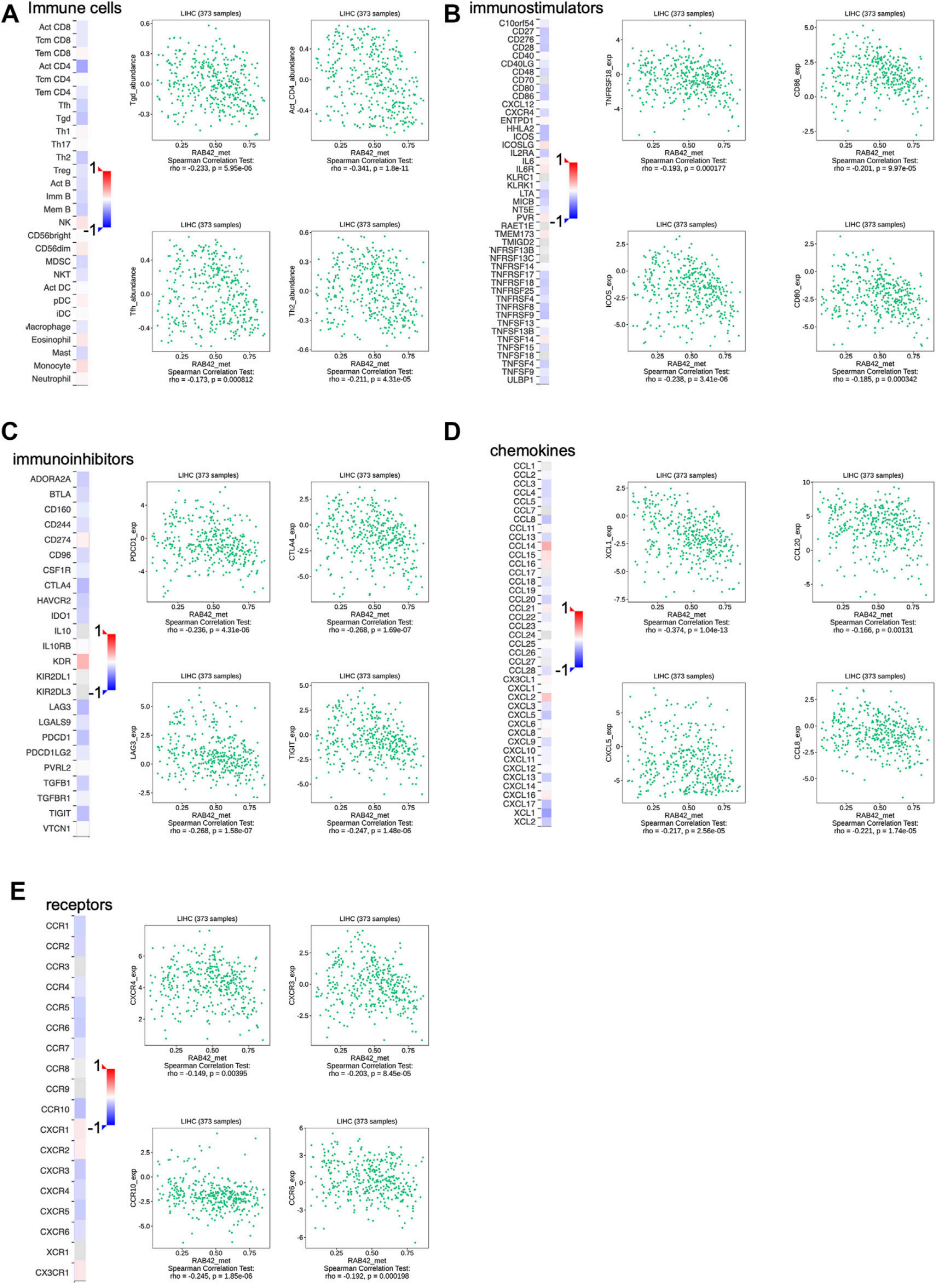
Association between the methylation level of RAB42 with immune cells infiltration in HCC. **(A)** Heatmap showed the correlation of RAB42 methylation status with immune-infiltrating lymphocytes in HCC. RAB42 methylation status was negatively associated with gdT helper cells, Activated CD4^+^ T, T follicular helper cells, and Type 2 T helper cells in HCC from the TISIDB database. RAB42 methylation status was negatively correlated with immune checkpoint molecules (TNFRSF18, CD86, ICOS, CD80) **(B)**, immunoinhibitors (PDCD1, CTLA4, LAG3, TIGIT) **(C)**, chemokines (XCL1, CCL20, CXCL5, CCL8) **(D)** and receptors (CXCR4, CXCR3, CCR10, CCR6) **(E)** in HCC from the TISIDB database.

Based on the analysis of the above results, RAB42 methylation level was related to the prognosis of HCC patients, we next assessed the correlation between RAB42 methylation and immune infiltration using TISIDB database. The results revealed that the methylation status of RAB42 was negatively correlated with Tgd cells (R = −0.233, *p* = 5.95e−06), activated CD4+T cells (R = −0.341, *p* = 1.8e−11), Tfh cells (R = −0.173, *p* = 8.12e−04) and Th2 cells (R = −0.211, *p* = 4.31e−05) (Figure 9A). Moreover, RAB42 methylation was negatively associated with immunostimulators, such as TNFRSF18 (R = −0.193, *p* = 1.77e−04), CD86 (R = −0.201, *p* = 9.97e−05), ICOS (R = −0.238, *p* = 3.41e−06) and CD80 (R = −0.185, *p* = 3.42e−04) (Figure 9B), meanwhile being negatively related with immune checkpoint molecules, such as PDCD1 (R = −0.236, *p* = 4.31e−06), CTLA4 (R = −0.286, *p* = 1.69e−07), LAG3 (R = −0.268, *p* = 1.58e−07) and TIGIT (R = −0.247, *p* = 1.48e−06) (Figure 9C). In addition, RAB42 methylation was also negatively correlated with chemokines and receptors, such as XCL1 (R = −0.374, *p* = 1.04e−13), CCL20 (R = −0.166, *p* = 0.00131), CXCL5 (R = −0.217, *p* = 2.56e−05), CCL8 (R = −0.221, *p* = 1.74e−05), CXCR4 (R = −0.149, *p* = 0.00395), CXCR3 (R = −0.203, *p* = 8.45e−05), CCR10 (R = −0.245, *p* = 1.85e−06) and CCR6 (R = -0.192, *p* = 1.98e-04) (Figures 9D,E). Overall, these results revealed that the RAB42 methylation was negatively correlated with immunosuppressive microenvironment in HCC.

### RAB42 Knockdown Attenuates the Proliferation, Migration, and Invasion of Hepatocellular Carcinoma

**FIGURE 10:**
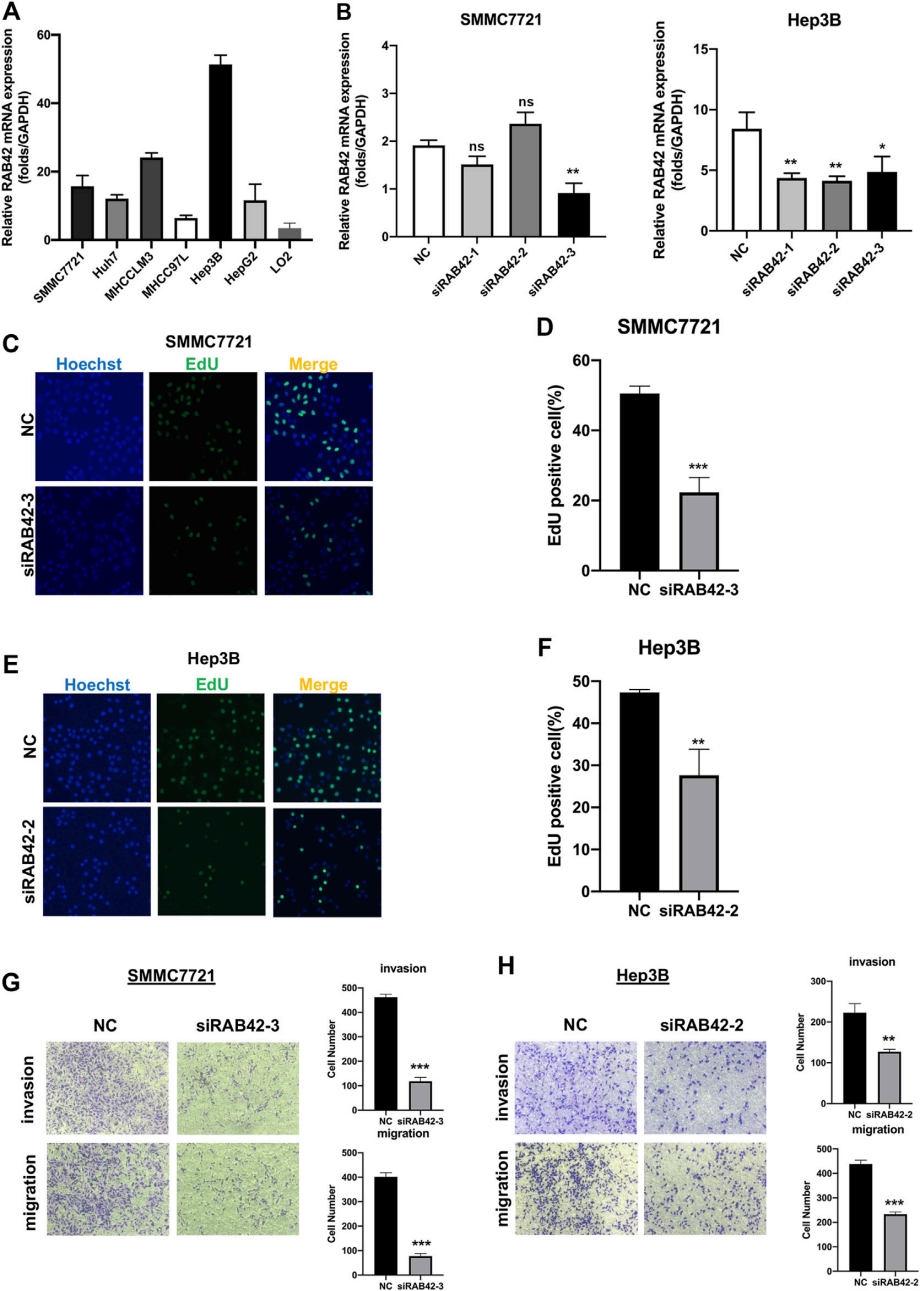
Knockdown of RAB42 inhibited the proliferation, invasion, and migration of liver cancer cells. **(A)** qRT-PCR assay was performed to compare the mRNA expression level of RAB42 in different liver cancer cell lines, including SMMC7721, Huh7, MHCCLM3, MHCC97L, Hep3B, HepG2 and normal hepatic cell line, LO2. *n* = 3. **(B)** qRT-PCR assay was performed to detect the silencing efficiency of RAB42 by three different siRNA in SMMC772 cells and Hep3B cells. Data were presented as the mean ± SD, *n* = 3, *p < 0.05, **p < 0.01. ns, no significance; siRNA, small interfering RNA; NC, negative control. **(C** **,** **D)** Representative images of the EdU assay of SMMC772 cells transfected with siRAB42-3 and quantitative measurement of the proportion of EdU-positive SMMC7721 cells. Silencing RAB42 expression decreased the proliferation of SMMC7721 cells. Data were presented as the mean ± SD, *n* = 3. ***p < 0.001. **(E** **,** **F)** Representative images of the EdU assay of Hep3B cells transfected with siRAB42-2 and quantitative measurement of the proportion of EdU-positive Hep3B cells. Silencing RAB42 expression decreased the proliferation of Hep3B cells. Data were presented as the mean ± SD, *n* = 3. **p < 0.01. **(G** **,** **H)** Transwell assays showed the invasion and migration of SMMC7721 cells **(G)** and Hep3B cells **(H)** transfected with or without siRAB42. Silencing RAB42 expression inhibited the invasion and migration of SMMC7721 and Hep3B cells. Data were presented as the mean ± SD, *n* = 3. **p < 0.01, ***p < 0.001.

To verify the role of RAB42 in HCC *in vitro*, we first analyzed RAB42 mRNA expression levels in liver cancer cell lines, including SMMC7721, Huh7, MHCCLM3, MHCC97L, Hep3B, HepG2 and normal hepatic cell line, LO2 (Figure 10A). The results confirmed that RAB42 was highly expressed in liver cancer cells compared with normal liver cells. Therefore, we selected two liver cancer cell lines, SMMC7721 and Hep3B cells with relatively high RAB42 expression, for subsequent knockdown experiments. SMMC7721 and Hep3B cells were transfected with three different siRNA of RAB42. We found that siRAB42-3 showed the highest knockdown efficiency in SMMC7721 cells, while siRAB42-2 showed the highest knockdown efficiency in Hep3B cells (Figure 10B). We used these two siRNAs separately in subsequent experiments. To explore the effect of RAB42 on the proliferation ability of liver cancer cells, an EdU assay was performed. The results showed the proportion of EdU-positive cells was significantly decreased with the transfection of siRAB42 in SMMC7721 and Hep3B cells, indicating that knockdown of RAB42 significantly inhibited the proliferation of liver cancer cells (Figures 10C–F). Then, transwell assays were performed to detect the role of RAB42 expression in regulating the invasion and migration of liver cancer cells. The results showed that silencing RAB42 expression decreased the invasion and migration ability of SMMC7721 and Hep3B cells (Figures 10G,H). Collectively, these results indicated that RAB42 was involved in the regulation of the proliferation, invasion, and migration of liver cancer cells *in vitro*, and the specific molecular mechanism needs to be further explored in the future.

## Discussion

HCC is the second most frequent cancer-related death worldwide, most HCC patients are diagnosed at an advanced stage, the treatment options for these advanced patients are limited (Chen et al., 2020). In the past few years, increasing studies have shown the pivotal role of the TME in tumor progression. The intercommunication between various immune-infiltrating lymphocytes and tumor cells constructs an immunosuppressive microenvironment that favors tumor growth. Recently, immunotherapy has arisen as a promising approach for treating HCC in clinical trials, however, a considerable proportion of patients are not sensitive to this treatment (Hernandez-Gea et al., 2013). Therefore, exploring immune-related genes may help for enhancing the antitumor immune response and inhibiting the immune escape of the tumor, thereby killing tumor cells to restrain tumor growth.

RAB42, also known as Rab7B, is a member of the Rab family of small GTPases and is associated with protein degradation activity (He et al., 2020). The previous study has reported that RAB42 expression was negatively correlated with overall survival in glioma and could be used as a predictor for the prognosis of patients with glioma (Zhang et al., 2019). In addition, *in vitro* experiments also validated that RAB42 also promoted the proliferation and invasion of glioma cells, which may be related to the activation of the VEGF pathway (Liu B. et al., 2021). However, the research of RAB42 in HCC is lacking, so we aimed to detect the expression levels of RAB42 in HCC, and to explore the relationship between RAB42 and DNA methylation status and immune cells infiltration of liver cancer by employing available databases.

In this study, we identified that RAB42 has a higher expression level in HCC tissues compared to normal tissues. Moreover, the RAB42 expression remarkably correlated with tumor stage, histologic grade, and patients’ age, and the advanced HCC patients tend to have higher expression of RAB42. In addition, HCC patients who have higher RAB42 expression had poorer OS, PFS, and DSS. Univariate Cox analysis showed that clinical stage, T stage, M stage, and RAB42 expression all have considerable value to predict the outcomes of HCC patients. Regrettably, the multivariate Cox analysis showed no statistical difference in RAB42 expression, suggesting that RAB42 is not yet an independent prognostic factor for the HCC cohort.

Previous research has revealed that genetic alterations accumulated in HCC were numerous, and specific genetic alterations may promote tumorigenesis (El Tekle et al., 2021), so we investigated whether RAB42 mutation may play a critical role in hepatocarcinogenesis. The percentage of RAB42 genetic alteration in HCC was about 5%, and the genetic alteration showed significant association with a poor OS and DFS. In addition to genetic changes that promote tumor growth, epigenetic changes also affect tumor progression and prognosis (Esteller, 2007). One of the major epigenetic processes that promote HCC tumorigenesis is an aberrant DNA methylation status (Baylin, 2002). Comprehensive genome analysis also revealed that high-frequency mutation of genes may be caused by hypomethylation modification of the gene promoter region (Hama et al., 2018). In HCC, hypomethylation was found to activate proto-oncogene and influence the prognosis of HCC (Calvisi et al., 2007). The relationship between DNA methylation levels of RAB42 and the prognosis of HCC patients was investigated. We found that the promoter methylation levels of RAB42 in HCC tissues were significantly lower than that in normal samples, suggesting that a low level of the promoter methylation status of RAB42 may contribute to the overexpression of RAB42 in HCC. Furthermore, two CpG sites, cg03757398, and cg04896949, were correlated with a worse OS in HCC. These data suggested that RAB42 hypomethylation was an effective predictor for the poor prognosis of HCC patients.

Few studies have investigated the role of RAB42 in tumors, to reveal the potential biological function of RAB42 in HCC, we searched the genes that co-expressed with RAB42 in liver cancer, and the functional enrichment analysis on these co-expressed genes was performed. Notably, the KEGG results showed that most of the genes co-expressed with RAB42 were involved in immune-related diseases, including allograft rejection, autoimmune thyroid disease and graft-versus-host disease, and immune-related processes, such as T cell/B cell receptor signaling pathway, Th1 and Th2 cell differentiation and natural killer cells mediated cytotoxicity. Similarly, GO analysis revealed that RAB42 may be involved in the activation of immune cells in HCC, for instance, mast cell activation, T/B cell activation, and myeloid dendritic cell activation. These results preliminarily suggested that RAB42 may be involved in immune regulation in HCC.

Tumor-infiltrating immune cells in the tumor microenvironment are divided into tumor-promoting immune cells and anti-tumor immune cells (Hiraoka, 2010). During tumor progression, high expression of specific oncoproteins in tumor cells may recruit immunosuppressive lymphocytes, such as MDSCs (Taki et al., 2018), regulatory T cells (Tregs) (Lu et al., 2021), and enhance their immunosuppressive function, or make anergy of tumor-killing lymphocytes such as CD8^+^ T cells and NK cells (Crespo et al., 2013; Judge et al., 2020), creating an ecological environment suitable for tumor growth. To elucidate the role of RAB42 in the TME, the relationships between RAB42 and immune infiltration in HCC were analyzed by TIMER and TISIDB databases. Our study suggested that RAB42 transcriptional expression positively correlated with various immune cell populations, including B cells, CD8^+^ T cells, CD4^+^ T cells, macrophages, neutrophils, and DCs in HCC. Furthermore, GEPIA database analysis showed RAB42 was also positively correlated with biomarkers of these infiltrated immune cells. Previous studies found that CD206 + M2 macrophage cells and Treg cells exert immune suppression function in the TME of HCC (Allavena et al., 2008), our study found that RAB42 may be involved in the recruitment of these immunosuppressive immune cells into the TME, which needs further experimental verification in the future. Accumulating evidence have shown that chemokines and chemokine receptors are closely related to the immunity of HCC. RAB42 transcriptional expression was positively correlated with immune chemokines such as CCL22 and CCL2, which can be secreted by tumor cells to recruit the Tregs and TAMs respectively into the TME (Li X. et al., 2017; Gao Y. et al., 2022). Interestingly, RAB42 was also positively associated with infiltration of antitumor immune cells such as CD8^+^ T cells and NK cells, which suggested that RAB42 has dual regulatory effects on immune cell infiltration during hepatocarcinogenesis. Due to the TME of HCC is immunosuppressive, when continuously exposed to tumor antigens, effector T cells may be in an exhausted state, causing the inefficiency of anti-tumor function. Expression of multiple immune checkpoint molecules is a common feature of exhausted T cells (Wherry, 2011). Our results showed that RAB42 was positively correlated with the expression of these checkpoint molecules in HCC, such as PDCD1, CTLA-4, LAG-3, HAVCR2, TNFRSF18, and TIGIT, thereby promoting T cells anergy. Another study showed that both IL-10 and TGF-β in TME can attenuate or inhibit the activation of immune cells (Ejrnaes et al., 2006; Tinoco et al., 2009). By using the GEPIA database, we found that RAB42 was positively correlated with IL10 and TGF-β in HCC. In addition, CD4^+^ Treg cells have immunosuppressive properties that inhibit the activation and proliferation of effector T cells, thereby promoting the exhaustion of T cells (Gao R. et al., 2022). By using various algorithms in the Timer database, we found that RAB42 was also positively correlated with the infiltration of Treg cells. A newly published article showed that exhausted CD8^+^ T cells can be divided into four stages, namely T cell exhaustion progenitors 1 (Tex Prog1), T cell exhaustion progenitors 2 (TexProg2), T cell exhaustion intermediate (TexInt), and T cell exhaustion terminally (Tex Term) (Beltra et al., 2020), we analyzed the association between RAB42 and the main genes in different stages by using GEPIA datasets. The results showed that RAB42 was positively correlated with exhausted CD8^+^ T cells in each stage. Overall, the above results indicated that although RAB42 recruit the infiltration of anti-tumor immune cells, it could contribute to the formation of the immunosuppressive milieu by stimulating the expression of immune checkpoint molecules, the production of Treg cells and immunosuppressive cytokines, inducing this group of effector cells in a non-functional exhausted state. Meanwhile, it may also indicate that targeting RAB42 might increase the efficacy of immune checkpoint inhibitors in HCC.

Apart from the infiltrating immune cells, as the main component of tumor stroma, CAFs participated in the formation of TIME and were involved in the tumor progression and prognosis in HCC (Jia et al., 2021). RAB42 expression was found to be significantly correlated with CAFs in HCC. Moreover, our results also found that RAB42 expression was positively correlated with specific markers of CAFs, such as a-SMA, FAP, Vimentin, and FSP1. Activated CAFs exhibit enhanced ECM synthesis properties, such as producing various types of collagens, hyaluronan, fibronectins, and laminins (Rodemann et al., 1991; Tomasek et al., 2002). We also found RAB42 expression was associated with stroma-related gene markers, for instance, FLNA, ELN, COL1A1, COL1A2. These results also implied that the RAB42 expression may induce the infiltration of CAFs to promote the immune escape of tumor cells and exacerbate the formation of TIME.

Accumulating studies have shown that epigenetic modifications in tumor cells, such as DNA methylation, play an important role in regulating TIL function, resulting in immune escape in the TME (Roulois et al., 2015; Hogg et al., 2020). Recent studies have shown that DNA methylation modification can promote the production of immune-related cytokines to promote tumor progression (Peng et al., 2015). Understanding how DNA methylation affects the tumor immune microenvironment provides a new perspective for combination immunotherapy. To explore the regulatory role of RAB42 methylation status on immune infiltration in the TME, the relationship between the methylation status of RAB42 and immune cells, immunomodulators, chemokines and receptors were analyzed. Our results showed that the RAB42 methylation negatively correlated with various immune cells, immune checkpoint molecules, immunostimulatory factors, most chemokines, and receptors. These results suggest that hypomethylation of RAB42 may recruit immunosuppressive cells and induce T cell exhaustion, while possibly promoting the production of immunosuppressive cytokines to contribute to an immunosuppressive milieu in HCC. Collectively, the methylation of RAB42 may be used as an indicator of tumor immune infiltration and targeting RAB42 may be a strategy to improve the efficiency of checkpoint inhibitor drugs.

Based on the above bioinformatics mining, the effect of RAB42 on the biological functions in HCC was also explored *in vitro*, such as proliferation, invasion and migration. We knocked down RAB42 through transfection siRNAs in two HCC cell lines with high RAB42 expression (SMMC7721 and Hep3B). The results showed that silencing RAB42 impaired proliferation, invasion, and migration of HCC cells. Overall, these results suggested that RAB42 is pivotal for maintaining the tumorigenic activity of HCC cells *in vitro*. However, it is required to further explore the specific mechanism and function of transcriptional expression/DNA methylation of RAB42 in regulating TME of HCC.

## Conclusion

In conclusion, this study revealed that RAB42 expression was associated with multiple clinical characteristics, meanwhile, its expression and methylation status correlated with immune cells infiltration and the prognosis of HCC. Moreover, *in vitro* experiments indicated that RAB42 promoted the proliferation, migration, and invasion of HCC. Therefore, our research highlights that RAB42 may be a novel immunomodulatory molecule in HCC. Further mechanistic studies and *in vivo* experiments are needed to validate our findings and promote the clinical application.

## Data Availability

The datasets presented in this study can be found in online repositories. The names of the repository/repositories and accession number(s) can be found in the article/Supplementary Material.

## Supplementary Material

Supplementary Figure S1
**(A)** RAB42 expression was positively correlated with IL10 and TGFB1 expression in HCC based on the GEPIA database. **(B)** The correlation between RAB42 expression and the infiltration level of Tregs was investigated by using various algorithms in HCC from the TIMER database. Tregs, regulatory T cells. **(C)** The table summarized the correlation coefficients R and *p* values of RAB42 and major genes of various stages of T cell exhaustion in HCC based on the GEPIA database.
