# Supplementary figures and images for: RAB42 is a Potential Biomarker that Correlates With Immune Infiltration in Hepatocellular Carcinoma
Source: Front Mol Biosci. 2022 May 26;9:898567. doi: 10.3389/fmolb.2022.898567 (PMC9204584; doi:10.3389/fmolb.2022.898567)

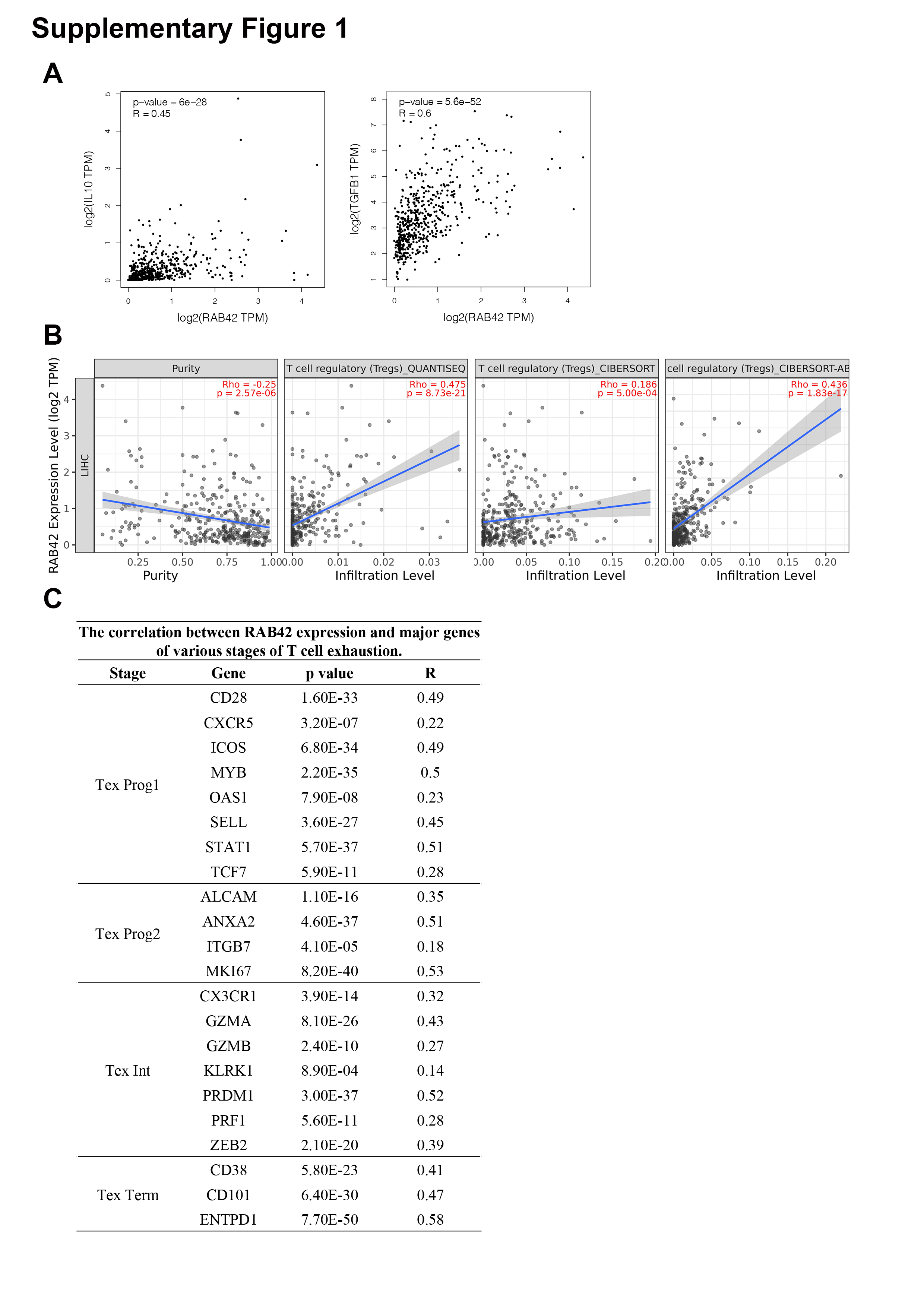

Supplement: Supplementary file 1 [file Image1.TIF]
